# Myeloid Sarcomas Causing Unilateral Cranial Nerve Palsies in a Patient with Relapsed Acute Myeloblastic Leukemia

**DOI:** 10.1155/2020/3749565

**Published:** 2020-01-13

**Authors:** A. Mendez-Hernandez, X. A. Andrade, S. Upadhyay, L. M. Parra-Rodriguez, E. Caldeira, L. H. Paz, H. Mann, M. Zia, L. Sumoza

**Affiliations:** ^1^Department of Medicine, John H. Stroger Jr. Hospital of Cook County, Chicago, IL, USA; ^2^Department of Hematology and Oncology, Mayo Clinic, Rochester, MN, USA; ^3^Department of Medicine, Advocate Illinois Masonic Medical Center, Chicago, IL, USA; ^4^Department of Cardiology, North Shore University Hospital, Evanston, IL, USA; ^5^Department of Hematology and Oncology, Tufts Medical Center, Boston, MA, USA; ^6^Department of Hematology and Oncology, John H., Stroger Jr., Hospital of Cook County, Chicago, IL, USA; ^7^Department of Hematology and Oncology, Alton Memorial Hospital, Alton, IL, USA

## Abstract

Myeloid sarcomas (MS) are a rare manifestation of myeloid malignancies and can often be misdiagnosed, leading to a delay in treatment. The objective of this clinical case is to highlight the challenges of the clinical presentation and to emphasize the importance of this manifestation ensuring timely diagnosis and therapy. Here, we present a 43-year-old man who was diagnosed with acute myeloblastic leukemia (AML) after being evaluated for unintentional weight loss, subcutaneous nodules, thrombocytopenia, and anemia. The patient underwent chemotherapy with complete remission and presented 4 months later with dysphagia and cranial nerve palsies. Appropriate imaging and biopsy led to a diagnosis of myeloid sarcoma, and a decision was made to begin reinduction chemotherapy for AML achieving a second complete remission although his neurological deficits did not improve. Our case illustrates the protean presentation of myeloid sarcomas; clinicians should have a high suspicion for MS and remain vigilant when unexplained signs and symptoms arise in the background of a myeloid malignancy although challenges still remain when presentation is de novo. Advancements in understanding the pathophysiology of MS have been performed but remain not completely understood. High clinical suspicion, appropriate imaging, biopsy techniques, and expertise are paramount for timely diagnosis and treatment.

## 1. Introduction

Myeloid sarcomas (MS) are extramedullary tumors composed of malignant immature myeloid cells and represent a rare complication of myeloid malignancies. In patients with acute myeloblastic leukemia (AML), the prevalence of MS ranges from 3 to 9%, but certain AML subtypes may have a higher prevalence. Common sites of involvement are skin, soft tissue, bone, and lymph nodes. Clinical presentation depends fundamentally on the size and location of the mass and mechanical compression of nearby structures. MS are commonly diagnosed concurrently with a primary myeloid malignancy; however, they can occur in the absence of bone marrow involvement by myeloid neoplasms which makes diagnosis challenging.

We present a case of a patient with a diagnosis of AML who presented with numerous myeloid sarcomas at the time of the first relapse.

## 2. Case Description

The patient is a 43-year-old man who underwent evaluation for unintentional weight loss and several abdominal subcutaneous nodules. Initial laboratory workup showed an elevated LDH level (1,165 UI/L) and the presence of anemia, thrombocytopenia, and circulating myeloid blasts in peripheral blood. Initial bone marrow aspiration and biopsy showed infiltration by 47% myeloid blasts with an abnormal karyotype (46, XY t(2; 21; 8) (p13; q22; q22), del(9)(q13q22)) and positive for *AML1-ETO* fusion gene and *c-kit* mutation. Further molecular studies for CEBPA, FLT3, NPM, PML RARA, and BCR/ABL were negative for mutations, and myeloid immunophenotype was positive for CD34, MPO, CD117, and CD56. Computerized tomography (CT) of the chest, abdomen, and pelvis showed numerous soft-tissue masses involving anterior mediastinum, pericardium, and subcutaneous tissue in the abdomen and pelvis.

The patient was diagnosed with acute myeloblastic leukemia with t(8; 21) variant and extramedullary involvement. He underwent induction and consolidation chemotherapy attaining a complete remission.

**Figure 1:**
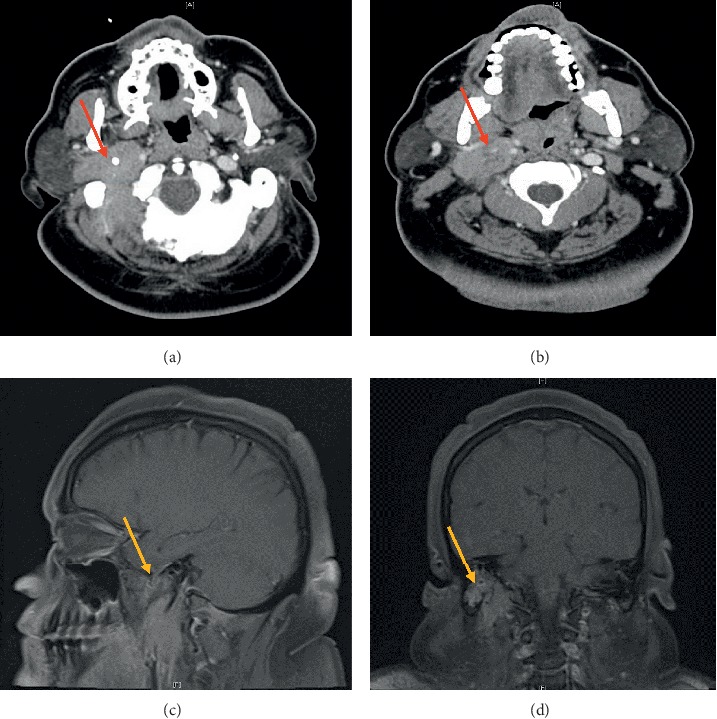
CT scan of the neck and soft tissue showing and infiltrative mass on the right skull base extending to the right jugular and stylomastoid foramen (a) encasing and occluding the right jugular vein (b). MRI confirms a soft-tissue mass on the skull base without intracranial involvement (c and d).

Four months after completion of therapy, the patient developed acute oropharyngeal dysphagia and right facial asymmetry. On physical examination, there was a right parotid gland enlargement and a palpable left axillary four-centimeter mass. Neurological exam was relevant for palsies of cranial nerves VII, X, and XII. Laboratory results were unremarkable, and a peripheral blood smear did not show leukemic blasts. CT and magnetic resonance imaging (MRI) of the neck and soft tissue showed an infiltrative soft-tissue right skull base mass extending into the inferior margin of the right jugular foramen, right stylomastoid foramen, deep and superficial lobes of the right parotid gland, the right temporomandibular junction, and styloglossus muscle also causing displacement of the internal carotid artery, encasing the internal jugular vein to the point of occlusion.  Figure 1.

**Figure 2:**
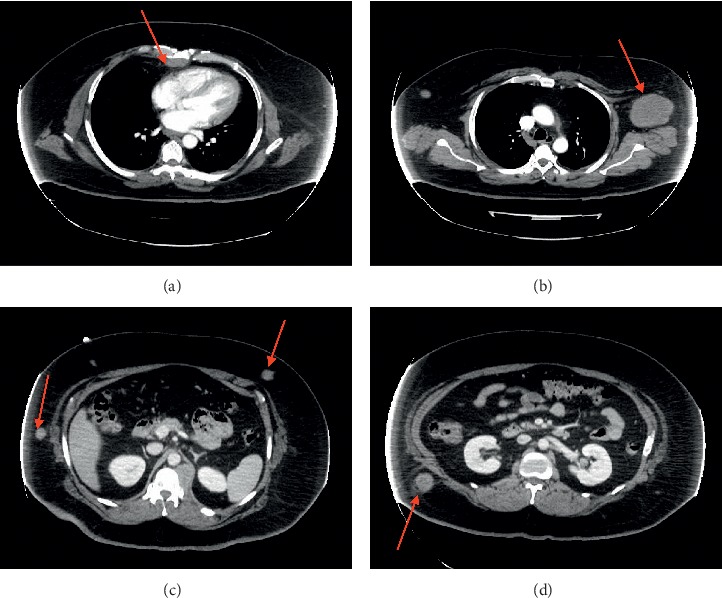
CT scan of the chest and abdomen showing multiple soft-tissue masses involving the anterior mediastinum (a), left axillary, (b) and abdominal subcutaneous tissue (c and d).

**Figure 3:**
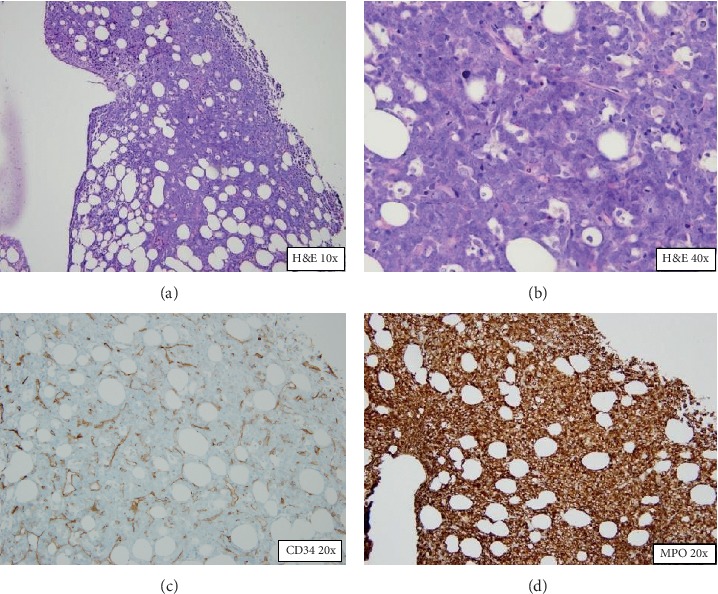
Bone marrow biopsy showed a hypercellular bone marrow with a diffuse infiltrate of large myeloid blast cells staining positive for CD34 and MPO markers in immunohistochemistry studies.

**Figure 4:**
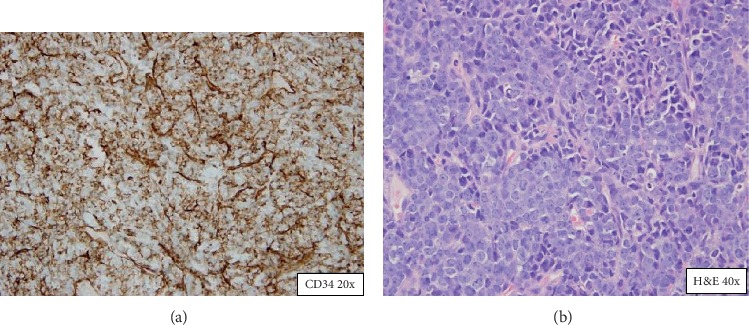
Core-needle biopsy shows a large atypical monomorphous infiltrate staining strongly for CD34 and MPO diffusely.

CT scan of the chest, abdomen, and pelvis showed multiple soft-tissue masses in the anterior mediastinum (4 × 1.5 cm), left renal hilum (1.7 × 1.9 cm), left paraspinal area (3.6 × 4 cm), and subcutaneous tissue in the abdomen and pelvis Figure 2. Biopsy of the bone marrow and subcutaneous left axillary mass confirmed a relapse of acute myeloblastic leukemia and myeloid sarcoma, respectively. Figures 3 and 4. The patient underwent reinduction chemotherapy attaining a second complete response of medullary and extramedullary disease and is currently receiving consolidation chemotherapy. Despite the complete remission of his leukemia, his neurological deficits did not resolve.

## 3. Discussion

MS is a rare extramedullary tumor of immature granulocytic cells or myeloblasts, frequently associated with myeloid hematological malignancies. In patients with AML, the prevalence of MS ranges from 2% to 8% of cases, either as a single or as a multifocal tumor [1, 2]. Interestingly, MS can precede AML by months or years in 25%, appear concomitantly in 15–35%, or follow AML in 50% of patients [1].

Patients with AML may have a different prevalence of MS based on French-American-British (FAB) classification subgroups. In large case series, the most common FAB subtype associated with MS was M5 (14%) [3], possibly demonstrating a relationship between morphologic phenotype, underlying cytogenetic and molecular abnormalities frequently associated with MS.

Cytogenetic and molecular abnormalities are present in 55% of cases of MS [4, 5, 6], the most common being t(8; 21), inv(16), and trisomy 8 [7]. Interestingly, patients with AML and t(8; 21) have a high prevalence of MS ranging from 2–30% among different studies [3, 5, 8]. C-kit proto-oncogene mutations are also common, appearing up to 87% of cases of MS [6] The clinical relevance of cytogenetic and molecular aberrancies is still under investigation.

Pathogenesis of MS is not completely understood; however, molecular interactions between malignant cells and their microenvironment is thought to be the main component. Animal studies in murine models by Stefanidakis et al. suggest a mechanism involving a supramolecular complex of matrix metalloproteinases and integrins (MMP-integrin), referred to as “*invadosome*” [9]. The interaction between the invadosome and leukocyte surface B2 integrin could mediate pericellular proteolysis and migration of AML-derived cells into extramedullary tissues [9]. Other adhesion molecules as CD56 have also been associated with higher frequency of MS; however, the exact mechanism involving this molecule is not fully understood [10, 11].

Clinical presentation depends on size and location of MS; signs and symptoms develop mainly from compression of adjacent structures due to mass effect, distorting normal organ architecture and causing pain and/or organ dysfunction. In a large case series by Pileri et al., the most commonly involved sites were the skin (28.2%), lymph nodes (16.3%), testis (6.5%), and intestines (6.5%) [3].

Patients with myeloid hematological malignancies presenting with soft-tissue masses can present a diagnostic challenge given the broad differential diagnoses including MS, infectious causes, and second primary malignancies such as melanoma or Hodgkin's lymphoma [12]. Unfortunately, up to 56% of patients of AML-related MS and up to 40% of de novo MS are misdiagnosed [2, 13]. Common misdiagnoses are diffuse large B-cell lymphoma (20%), myeloid metaplasia (4%), and small lymphocytic lymphoma (4%) [3]. Examination by an experienced pathologist and immunohistochemistry testing is paramount for accurate diagnosis.

Standard induction chemotherapy is the largely accepted treatment for AML-related MS. However, there is much debate regarding patients with isolated MS. There are currently no guidelines, but it is agreed that patients with isolated MS should receive chemotherapy given that they almost always progress to AML in a median of 5–12 months [10, 13]. Treatment strategies for relapsed AML presenting as MS vary depending on prior chemotherapy. Overall, current expert opinion recommends reinduction chemotherapy and radiation therapy if relapse occurs after chemotherapy alone [10]. Consolidation treatment with allogeneic bone marrow transplant has shown to prolong survival and may be recommended in relapsed patients [3]. The prognostic relevance of MS remains unclear, but it is widely considered as a poor prognostic factor in patients with AML [10, 14].

Future directions in patients with MS include investigation of the molecular mechanisms of disease and development of risk-stratification models to guide decisions regarding risk-adapted treatment interventions including bone marrow transplantation.
